# Disparities in Social Determinants of Health Among Patients Receiving Liver Transplant: Analysis of the National Inpatient Sample From 2016 to 2019

**DOI:** 10.7759/cureus.26567

**Published:** 2022-07-05

**Authors:** Mahmoud M Mansour, Darian Fard, Sanket D Basida, Adham E Obeidat, Mohammad Darweesh, Ratib Mahfouz, Ali Ahmad

**Affiliations:** 1 Internal Medicine, University of Missouri School of Medicine, Columbia, USA; 2 Internal Medicine, University of Hawaii, Honolulu, USA; 3 Internal Medicine, East Tennessee State University, Johnson City, USA; 4 Internal Medicine, Kent Hospital/Brown University, Warwick, USA; 5 Gastroenterology and Hepatology, University of Missouri School of Medicine, Columbia, USA

**Keywords:** socio-demographic disparity, race inequities, gender equity, nationwide inpatient sample (nis), liver transplant, social determinants of health (sdoh), health care disparities

## Abstract

Background

Liver transplantation is the life-saving standard of care for those with end-stage liver disease. Unfortunately, many patients on the liver transplant list die waiting. Several studies have demonstrated significant differences based on disparities in race, gender, and multiple socioeconomic factors. We sought to evaluate recent disparities among patients receiving liver transplants using the latest available data from the National Inpatient Sample (NIS), the largest publicly available inpatient care database in the United States.

Methods

We performed an analysis of discharge data from the NIS between 2016 and 2019. We identified adult patients with chronic liver disease who underwent a liver transplant using the International Classification of Diseases, 10th revision (ICD-10) codes. Multivariate logistic regression was used to adjust for differences in race, gender, socioeconomic status, and comorbidities among those who received a liver transplant.

Results

A total of 24,595 liver transplants were performed over the study period. Female gender was independently associated with decreased transplant rates (adjusted odds ratio (AOR) 0.83, 95% confidence interval (CI), 0.78-0.89, P < 0.001). Compared to White patients, Black patients had decreased transplant rates (AOR 0.86, 95% CI, 0.75-0.99, P = 0.034), as did Native Americans (AOR 0.64; 95% CI, 0.42-0.97, P = 0.035). Hispanics and Asian Americans had increased rates of liver transplantation (AOR 1.16, 95% CI 1.02-1.32, P = 0.022, and 1.36, 95% CI 1.11-1.67, P = 0.003; respectively). The increase in income quartile was associated with an incremental increase in transplant rates. Additionally, patients with private insurance had much higher transplant rates compared to those with Medicare (AOR 2.50, 95% CI 2.31-2.70, P < 0.001) while patients without insurance had the lowest rates of transplantation (AOR 0.18, 95% CI 0.12-0.28, P < 0.001).

Conclusions

Our analysis demonstrates that race, gender, and other social determinants of health have significant impacts on the likelihood of receiving a liver transplant. Our study, on a national level, confirms previously described disparities in receiving liver transplantation. Patient-level studies are needed to better understand how these variables translate into differing liver transplantation rates.

## Introduction

According to the Centers for Disease Control and Prevention (CDC), over 100,000 individuals in the United States died from cirrhosis or its complications between 2016 and 2019 [1]. Thousands of patients with end-stage liver disease die waiting for liver transplantation annually due to an overwhelming gap between organ demand and availability. Unfortunately, this gap is increasing every year [2]. Since 1984, the Organ Procurement and Transplantation Network (OPTN) has been working to ensure equal distribution and allocation of organs [3].

In 2002, the Model for End-Stage Liver Disease (MELD) score was validated in the United States to predict mortality in transplant patients with end-stage liver disease and prioritize patients for liver transplants accordingly [4]. The scoring system utilizes three clinical parameters: serum creatinine, serum bilirubin, and international normalized ratio (INR) to calculate a score of a total of 40 points. However, liver transplantation, similar to other fields of organ transplant in the United States, has not been equally available to all patients [5]. Epstein et al. found that Black patients only constituted 17% of patients who received renal transplantation compared to 52% of White patients [6]. Herring and colleagues found that the uninsured were much less likely to receive organ donation (0.8%) but comprised 16.9% of donor organs [7].

Several studies have discussed disparities in liver transplantation in previous years; however, there is a lack of literature on more current trends. The purpose of our study was to provide an updated review of the many disparities previously described. We hypothesized that despite the many advances in medical care and treatment of patients with advanced liver disease, various disparities still exist. Therefore, we used the most recently available data set from the National Inpatient Sample (NIS) to analyze the extent of these disparities in liver transplantation.

## Materials and methods

Data source

We performed a cross-sectional analysis of NIS data from January 1, 2016, to December 31, 2019. The NIS is drawn from all states participating in the Healthcare Cost and Utilization Project (HCUP), a family of healthcare databases that cover more than 97% of the United States population. The NIS includes a sample of 20% of US hospitalizations that are subsequently weighted to be nationally representative of all US hospitalizations [8]. The database includes information on patient demographics, hospital characteristics, hospital outcomes, and up to 40 diagnostic and 25 procedure codes based on the International Classification of Diseases 10th revision, Clinical Modification (ICD-10-CM), and Procedure Coding System (ICD-10-PCS).

Study design and inclusion criteria

We identified adults aged 18 or older diagnosed with chronic liver disease (cirrhosis, regardless of etiology, and hepatocellular carcinoma) using ICD-10-CM codes. Subsequently, among those patients with chronic liver disease, patients who underwent liver transplantation were identified using the ICD-10-PCS codes. The demographic variables were age, gender, race, insurance (Medicare, Medicaid, private insurance, and no insurance), income quartile, hospital size (relative to location and teaching status as shown in Table 1 [9]), and hospital region. Medicare is an insurance program for people over 65, younger disabled people, and dialysis patients and Medicaid is an assistance program for low-income patients' medical expenses [10]. Patients missing any of the demographic variables were excluded.

**Table 1:** Bed size categories in the National Inpatient Sample according to the hospital's location and teaching status Source: [9]

Location and Teaching Status	Hospital Bed Size		
	Small	Medium	Large
Northeast region			
Rural	1-49	50-99	100+
Urban, nonteaching	1-124	125-199	200+
Urban, teaching	1-249	250-424	425+
Midwest region			
Rural	1-29	30-49	50+
Urban, nonteaching	1-74	75-174	175+
Urban, teaching	1-249	250-374	375+
Southern region			
Rural	1-39	40-74	75+
Urban, nonteaching	1-99	100-199	200+
Urban, teaching	1-249	250-449	450+
Western region			
Rural	1-24	25-44	45+
Urban, nonteaching	1-99	100-174	175+
Urban, teaching	1-199	200-324	325+

ICD-10-CM codes used in this study were C220 (liver cell carcinoma), K703 (alcoholic cirrhosis of the liver), K717 (toxic liver disease with fibrosis and cirrhosis of the liver), K742 (hepatic fibrosis with hepatic sclerosis), K766 (portal hypertension), K767 (hepatorenal syndrome), K721 (chronic liver failure). The ICD-10-PCS codes used in this study were 0FY00Z0 (Allogenic liver transplant) and 0FY00Z1 (Syngeneic liver transplant).

Statistical analysis

We used HCUP published recommendations for analysis using survey data with sampling weights to generate nationwide estimates [11]. We utilized chi-square analyses for categorical data. Subsequently, we utilized multivariable logistic regression to identify the independent impact of demographic variables on receiving a liver transplant. We included the Charlson comorbidity index to adjust for the overall disease burden. We also adjusted for the presence of alcoholic cirrhosis diagnosis in the model. Statistical hypotheses were tested using P<0.05 as the level of statistical significance. The statistical analysis was performed using STATA software, version 17.0 (StataCorp., College Station, TX).

## Results

**Figure 1:**
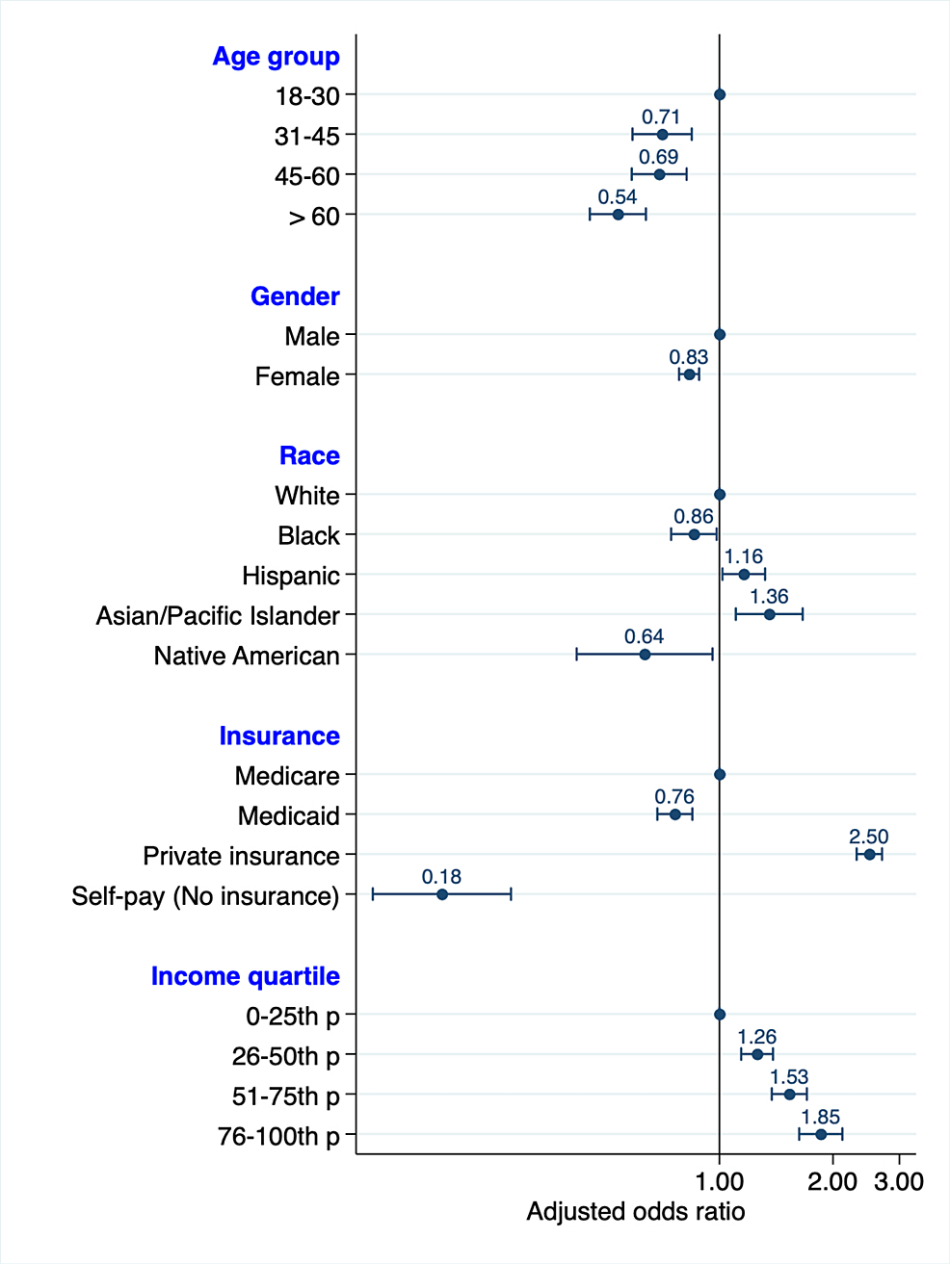
Adjusted odds ratio graph of the studied demographic variables Included in the multivariate analysis but not shown in this graph are the region of the hospital, Charlson comorbidity index, and alcoholic cirrhosis p: percentile

A total of 24,595 patients underwent liver transplantation from January 1, 2016, to December 31, 2019, in the United States. Table 2 details the number of patients diagnosed with chronic liver disease in each group and the proportion of those who underwent a liver transplant. All the studied demographic variables had statistically significant differences in transplantation rates except for the hospital region (P = 0.790). These differences were congruent with the adjusted findings in the multivariate analyses shown in Table 3. Figure 1 is a graphical representation of the study's demographic variables: age, gender, race, insurance, and income.

**Table 2:** Patients with chronic liver disease hospital diagnosis and the proportion and number of those who underwent a liver transplant *Chronic liver disease includes cirrhosis, regardless of the etiology, and hepatocellular carcinoma.

Demographic variable	Number of patients with chronic liver disease* diagnosis	Number of patients receiving transplant	Proportion of patients receiving a transplant	P-value
Gender				<0.001
Males	1,113,912	15,706	1.41%	
Female	766,441	8,891	1.16%	
Age group				<0.001
18-30	38,287	896	2.34%	
31-45	211,724	3,105	1.47%	
46-60	704,180	10,677	1.52%	
> 60	926,161	9,956	1.08%	
Race				<0.001
White	1,253,889	16,928	1.35%	
Black	187,745	1,840	0.98%	
Hispanic	303,964	3,739	1.23%	
Asian or Pacific Islander	47,092	928	1.97%	
Native American	30,324	200	0.66%	
Other				
Zip code income quartile				<0.001
1 - 47,999	610,979	5,804	0.95%	
48,000 - 60,999	496,703	5,960	1.20%	
61,000 - 81,999	442,204	6,589	1.49%	
82,000+	330,467	6,279	1.90%	
Payer				<0.001
Medicare	870,586	8,096	0.93%	
Medicaid	443,606	3,593	0.81%	
Private insurance	406,862	11,514	2.83%	
Self-pay (No insurance)	95,450	210	0.22%	
Hospital size				<0.001
Small	323,812	550	0.17%	
Medium	521,308	3,545	0.68%	
Large	1,035,234	20,498	1.98%	
Region of hospital				0.790
Northeast	326,401	4,243	1.30%	
Midwest	385,106	5,353	1.39%	
South	733,643	9,831	1.34%	
West	435,203	5,222	1.20%	

**Table 3:** The multivariate analysis model used in the study showing adjusted odds ratios for receiving a liver transplant for each variable along with 95% confidence intervals (CI)

Independent variables	Adjusted odds ratio	95% CI low	95% CI high	P-value
Gender				
Male	1 (reference)			
Female	0.83	0.78	0.89	<0.001
Age group				
18-30	1 (reference)			
31-45	0.71	0.59	0.85	<0.001
46-60	0.69	0.59	0.82	<0.001
> 60	0.54	0.45	0.64	<0.001
Race				
White	1 (reference)			
Black	0.86	0.75	0.99	0.034
Hispanic	1.16	1.02	1.32	0.022
Asian / Pacific Islander	1.36	1.11	1.67	0.003
Native American	0.64	0.42	0.97	0.035
Income				
1 - 47,999	1 (reference)			
48,000 - 60,999	1.26	1.14	1.39	<0.001
61,000 - 81,999	1.53	1.37	1.70	<0.001
82,000+	1.85	1.62	2.12	<0.001
Payer / Insurance				
Medicare	1 (reference)			
Medicaid	0.76	0.68	0.85	<0.001
Private insurance	2.50	2.31	2.70	<0.001
Self-pay (No insurance)	0.18	0.12	0.28	<0.001
Hospital size				
Small	1 (reference)			
Medium	3.87	2.11	7.10	<0.001
Large	11.78	6.98	19.90	<0.001
Region of hospital				
Northeast	1 (reference)			
Midwest	1.02	0.78	1.33	0.905
South	1.16	0.90	1.48	0.248
West	0.83	0.62	1.11	0.215
Charlson comorbidity index (for every one-point increase)	0.93	0.91	0.94	<0.001
Alcoholic cirrhosis	0.91	0.85	0.97	0.006

Gender disparities

There were 8,891 liver transplants in women (36.15%) and 15,706 in men (63.85%). On multivariate analysis, female patients had a lower adjusted odds ratio (AOR) of liver transplantation compared to male patients (AOR 0.83, 95% CI 0.78-.89, P < 0.001).

Age-related disparities

The older the patient, the less likely they were to receive liver transplantation. Patients aged 18 to 30 had the highest adjusted odds of receiving a liver transplant (AOR 1, reference group). In contrast, those above 60 had the lowest adjusted odds (AOR 0.54, 95% CI 0.45-0.64, P < 0.001).

Racial disparities

Our study showed that compared to White patients, Black and Native American patients were less likely to receive liver transplantation (AOR 0.86, 95% CI 0.75 - 0.99, P = 0.034 and AOR 0.66, 95% CI 0.49 - 0.97, P = 0.035, respectively). On the other hand, Hispanic and Asian patients were more likely to have liver transplant (AOR 1.16, 95% CI 1.02-1.32, P = 0.022 and AOR 1.36, 95% CI 1.11-1.67, P = 0.003; respectively).

Income and insurance disparities

In analyzing income-related factors, the rates of transplants increased incrementally with a higher average household income. Additionally, patients with private insurance, compared to patients on Medicare, had the highest likelihood of undergoing liver transplantation (AOR 2.50, 95% CI 2.31-2.70, P < 0.001). Patients without insurance had the lowest rates of transplantation (AOR 0.18, 95% CI 0.12-0.28, P < 0.001).

Disparities in hospital size and region

There were no statistically significant differences in liver transplantation rates across the different hospital regions. Expectantly, larger academic centers had higher rates of liver transplantation.

Disparities in medical comorbidities and etiology of liver disease

Medical comorbidities have a significant impact on liver transplantation rates. Patients with fewer or less severe comorbidities were more likely to undergo liver transplantation (for every one-point increase in Charlson comorbidity index, AOR decreased by 0.93, 95% CI 0.91-0.94, P < 0.001). In addition, patients with alcoholic cirrhosis diagnosis were less likely to receive liver transplantation (AOR 0.91, 95% CI 0.85-0.97, P = 0.006).

## Discussion

Our multivariate analysis reveals that there remain many independent variables that are associated with inequalities in receipt of liver transplants.

Females, compared to males, were less likely to receive liver transplantation at a national level. Ross-Driscoll et al. found a similar predominance of males in liver transplantation recipients (67.9%) compared to females in 2021 [12]. Mathur et al. found that females not only had lower liver transplantation rates (9%) in the pre-MELD era but also that this gender gap increased further (14%) in the post-MELD era [13]. Moylan et al. found that although waiting list mortality rates and delisting rates were similar, females had lower adjusted rates of transplantation, indicating that gender disparity possibly exists primarily in the enlisting process for liver transplants [14]. Many have conjectured the possible explanations for these findings. For instance, geographic variations in liver transplants among males and females have been shown by some studies [15]. This may support the argument that sex-biased practice in healthcare can vary in different geographic locations. It could also be due to reluctance in females in certain regions or cultural backgrounds to undergo or abstain from certain treatments or procedures [14-18]. Nonetheless, these gender-based differences open doors for further research.

There were marked differences in liver transplantation rates based on patient race. Compared to White patients, Black and Native American patients were less likely to receive liver transplants while Asians and Hispanic patients were more likely to. Multiple studies have shown similar racial disparities in other organ transplantation fields such as bone marrow, renal, and heart transplantation [19-25]. Interestingly, Moylan et al. previously demonstrated that race was not significantly associated with liver transplantation or death in the post-MELD era; however, they found that non-White patients were often listed at a more advanced stage of disease [14]. Prior institutional-level data from Julapalli et al. and Eckhoff et al. showed that Black and Hispanic patients were less likely to be listed [26-27]. Siegel et al. also found that Black patients were less likely to receive liver transplantation (adjusted OR 0.43) [21]. However, our study found that the adjusted OR for black patients was 0.86, suggesting some improvements in these disparities since 2008. Kemmer et al. found that Asians have better survival advantages compared to non-Asian groups and lower post-transplant mortality [27].

The mechanisms of how these disparities impact liver transplantation rates are likely multifactorial. For example, Lathan et al. highlighted that despite access to medical care, Black patients were less likely to undergo surgery for lung cancer. indicating that there might be a problem at the patient-physician level [28]. Some studies have shown that ABO blood grouping that varies by race may contribute to the availability of organ donors while others have shown that Blacks have a higher rate of alcoholic cirrhosis of the liver [29-30].

Furthermore, patients with higher Charlson comorbidity index scores were less likely to undergo transplantation. This is in concordance with work by Kanwal et al. who showed that, nationally, patients with more medical comorbidities had fewer referrals to transplant centers, were wait-listed more often, and were less likely to undergo liver transplantation [30]. Patients with alcoholic cirrhosis were less likely to undergo liver transplantation. Despite the lower rates of transplant in alcoholic cirrhosis patients, Lee et al. showed that between 2002 and 2016, patients who had alcoholic hepatitis had higher rates of referral to liver transplantation and higher rates of post-transplant survival [31]. Early identification of these patients, as well as timely referral, may allow for better outcomes for patients with alcohol-related liver disease.

Different transplantation rates based on insurance type and income level highlight some economic factors involved in the transplant process. These findings align with studies by Sarpel et al., who found that people on commercial insurance were almost twice as likely to receive a liver transplant for HCC than people on government insurance (i.e., Medicare and Medicaid) [32]. Bryce et al. and Yu et al. found similar socio-economic discrepancies in liver transplants [33-34]. Patients undergoing transplantation require comprehensive multidisciplinary care with hepatologists and surgeons and regular follow-ups with blood tests, imaging studies, and endoscopic procedures, all increasing the cost of transplantation. Unfortunately, this can become financially unrealistic for many patients and thus make it seem that patients with lower incomes are less appropriate candidates for liver transplantation.

Data from this study revealed that patients with cirrhosis secondary to alcohol abuse are less likely to receive transplants regardless of the other factors. Since many transplantation centers have a mandatory sobriety requirement for various periods of time, these patients likely have a higher risk of being deemed inappropriate candidates for liver transplantation [35]. Interestingly, work by Weeks et al. showed that in patients with alcoholic hepatitis, liver transplantation without abstinence for six months showed similar outcomes compared to those patients who received transplants only after six months of sobriety [36].

Disparities in access to liver transplantation are a significant focus in research worldwide. Eurotransplant countries (Austria, Belgium, Croatia, Germany, Hungary, Luxembourg, Netherlands, and Slovenia) have shown that females were less likely to be registered for liver transplants and more likely to be removed from the wait list [37]. Similar gender disparities were shown in other countries, such as Iran and China, where males constituted 61.5% and 83.7% of liver transplant recipients, respectively [38-39]. Socioeconomic factors have an impact on liver transplants globally as well. For example, in Taiwan, satellite and rural areas were associated with a lower prevalence of liver transplantation [40]. Similarly, in the United Kingdom, patients living >60 minutes from the nearest transplant center had a decreased chance of receiving or recovering without a transplant [41]. Further, in Iran, coverage of liver transplant costs by the Ministry of Health was effective in narrowing the gap between low and high socioeconomic classes [42].

A major strength of this study is the use of available nationwide data to assess current inequalities in liver transplantation. Previous studies have mainly resorted to using OPTN information from the United Network for Organ Sharing (UNOS), which is limited by no longitudinal follow-up, long-term outcomes, and the possibility that the patient sample may not be representative of the transplant population [43]. Despite the implementation of MELD scores 20 years ago and other criteria to help identify ideal candidates for liver transplantation, significant inequalities still exist across the nation. Another vital area of research would be analyzing data regarding recent trends in liver transplant listings and comparing them to prior trends.

Limitations 

The NIS lacks granular data, such as access to care, education, and social support systems, which have been shown to contribute to disparities in organ transplants [43]. These factors could help further elucidate the observed differences in receiving a liver transplant. Further, data included in the NIS is not sufficient to calculate the MELD score. This may limit our ability to understand the extent of those disparities among differing MELD scores.

Additionally, the nature of this study inherently does not allow for investigation at an individual level; the NIS data is at the hospitalization level. Furthermore, there may be more than one hospitalization per patient. Since we compared the proportions of liver transplants amongst hospitalizations with a diagnosis of advanced liver disease, it is unknown if different rehospitalization rates exist amongst demographic groups, which may have skewed the proportion of those receiving a transplant one way or the other. Nonetheless, the NIS is a large and nationally representative database that can be utilized as a reference tool for further patient-level research and potential implications on healthcare policies.

## Conclusions

Our analysis demonstrates that, at a national level, there remain disparities in recent years among patients with chronic liver disease who receive liver transplantation. These differences have persisted since before the MELD era, and their persistence in current times illustrates that more work remains ahead of us. Additional investigations at the patient level are needed to help understand the mechanisms by which these variables affect liver transplantation rates. Clinicians and transplant centers must acknowledge and address these disparities so that our community can progress to more equitable rates of liver transplantation.
